# Antiosteoporotic Nanohydroxyapatite Zoledronate Scaffold Seeded with Bone Marrow Mesenchymal Stromal Cells for Bone Regeneration: A 3D In Vitro Model

**DOI:** 10.3390/ijms23115988

**Published:** 2022-05-26

**Authors:** Matilde Tschon, Elisa Boanini, Maria Sartori, Francesca Salamanna, Silvia Panzavolta, Adriana Bigi, Milena Fini

**Affiliations:** 1 Complex Structure Surgical Sciences and Technologies, IRCCS Istituto Ortopedico Rizzoli, Via di Barbiano 1/10, 40136 Bologna, Italy; matilde.tschon@ior.it (M.T.); francesca.salamanna@ior.it (F.S.); milena.fini@ior.it (M.F.); 2 Department of Chemistry ‘‘Giacomo Ciamician”, University of Bologna, Via Selmi 2, 40126 Bologna, Italy; elisa.boanini@unibo.it (E.B.); silvia.panzavolta@unibo.it (S.P.); adriana.bigi@unibo.it (A.B.)

**Keywords:** bone fracture, in vitro model, human mesenchymal stromal cells, nanohydroxyapatite zoledronate scaffold, bone regeneration

## Abstract

Background: Bisphosphonates are widely employed drugs for the treatment of pathologies with high bone resorption, such as osteoporosis, and display a great affinity for calcium ions and apatitic substrates. Here, we aimed to investigate the potentiality of zoledronate functionalized hydroxyapatite nanocrystals (HAZOL) to promote bone regeneration by stimulating adhesion, viability, metabolic activity and osteogenic commitment of human bone marrow derived mesenchymal stromal cells (hMSCs). Methods: we adopted an advanced three-dimensional (3D) in vitro fracture healing model to study porous scaffolds: hMSCs were seeded onto the scaffolds that, after three days, were cut in halves and unseeded scaffolds were placed between the two halves. Scaffold characterization by X-ray diffraction, transmission and scanning electron microscopy analyses and cell morphology, viability, osteogenic differentiation and extracellular matrix deposition were evaluated after 3, 7 and 10 days of culture. Results: Electron microscopy showed a porous and interconnected structure and a uniform cell layer spread onto scaffolds. Scaffolds were able to support cell growth and cells progressively colonized the whole inserts in absence of cytotoxic effects. Osteogenic commitment and gene expression of hMSCs were enhanced with higher expressions of *ALPL*, *COL1A1*, *BGLAP*, *RUNX2* and *Osterix* genes. Conclusion: Although some limitations affect the present study (e.g., the lack of longer experimental times, of mechanical stimulus or pathological microenvironment), the obtained results with the adopted experimental setup suggested that zoledronate functionalized scaffolds (GHAZOL) might sustain not only cell proliferation, but positively influence osteogenic differentiation and activity if employed in bone fracture healing.

## 1. Introduction

Although bone is a highly dynamic tissue with innate and strong regenerative potential, large bone defects are very challenging in orthopaedic surgical practice. The defects can be due to malformation, traumatic events, bone resection caused by tumour or infection or from the treatment of complex non-unions [1]. The treatment of bone grafts or bone substitutes is currently used to solve critical bone defects; such surgical procedures encompass more than 10% of all skeletal reconstructive surgery cases, and worldwide, about 2–3 million grafting procedures are estimated each year [2,3].

Moreover, the presence of some patient-related conditions, such as ageing, malnourishment, oestrogen-depletion and impaired blood supply impedes the reparative biological events so that bone defects do not heal. Bone regenerative procedures in elderly patients or patients suffering of osteoporotic fractures are even more challenging, due to their poor mechanical and biological bone regenerative potentials [4]. The social and economic impact is huge since osteoporosis is the most common bone disease worldwide, affecting one in two women and one in five men, with estimated costs of around 37 billion EUR per year in the European Union (EU) [5,6]. Annually, osteoporotic fractures are more than 8.9 million worldwide, 3.5 million in the EU: about 17.7% of cases are located at the hip, 14.9% at the spine and 16% at the forearm. Pathological fractures cause a global loss of 5.8 million healthy life years to disability and are associated with increased mortality rate [7,8]. For example, hip fractures have a 30% mortality rate at 1 year and about half of patients are no longer able to live independently [9,10,11,12].

Beside these social and economic burdens, the use of biomaterials for prosthetic surgery, implantology or bone regenerative purposes has represented a great advance in reconstructive surgery, improving the quality of life of patients, relieving pain and allowing healthy ageing. Apart from natural bone derived substitutes, such as autografts and allografts, the most employed biomaterials have synthetic origins. On one hand, they show great mechanical properties, on the other hand, their biological performances such as osteoinduction, bioactivity and osteointegration with the recipient bone might be improved. So far, many efforts in the preclinical research and technological innovation have been focussed on their functionalization with porous or bio-active surfaces or coatings to balance biological and biomechanical and chemico-physical properties. The development of these multifunctional and multicomponent grafting biomaterials with bioactive molecules or materials, able to actively contribute to the regenerative/reparative processes, to promote the integration rate, to recruit cells, to stimulate biological processes, to hinder local inflammatory processes and to locally deliver drugs have gained much interest [13,14,15,16]. Implants and biomaterials intended for bone tissue should osteointegrate, where osteointegration is the histological and functional link between biomaterials and bone and is strictly dependent both from the biomaterial surface, geometry, chemistry and morphology and from the healing potential of the recipient bone [17].

In this context, much research by our group has been focused on the development of porous scaffolds as delivery systems for bisphosphonates to increase their local bioavailability [18]. To this aim, we enriched the composition of porous gelatin scaffolds with zoledronate functionalized hydroxyapatite nanocrystals (GHAZOL). Zoledronate is an amino bisphosphonate with powerful antiresorptive properties [19] and high binding affinity to hydroxyapatite (HA) [20]. The counteracting action of Zoledronate towards abnormally high bone resorption is maintained also when it is associated to hydroxyapatite: zolendronate doped HA has been demonstrated to promote osteoblast proliferation and differentiation, whereas it downregulates osteoclast viability and activity [21]. The amount of inorganic phase loaded on gelatin scaffolds, 30 wt%, was chosen based on the good values of porosity, connectivity and pore dimensions previously reported for this amount of hydroxyapatite nanocrystals [22].

However, from a preclinical point of view, there is the need for advanced innovative methods that might help scientists to investigate the potentialities of smart biomaterials and scaffolds.

In this scenario, we adopted, as a preclinical experimental platform, the 3D in vitro fracture healing model for studying wound healing, proposed by Sundelacruz and colleagues [23]. The aim of this study was to investigate the ability of an innovative scaffolding biomaterial doped with anti-osteoporotic drug (GHAZOL) in comparison with the bare undoped scaffold (GHA) to promote adhesion, viability, differentiation towards osteogenic phenotype and metabolic activity of hMSC. The 3D model was generated by differentiating hMSCs towards the osteogenic lineage on porous scaffolds that were subsequently “fractured” by cutting them in half in a cross-section. Then, two unseeded scaffolds were positioned between the two cut halves of the tissue to simulate the implantation of a scaffold into the defect. At selected experiment times, cell morphology, viability, osteogenic differentiation extracellular matrix deposition and the presence of inflammatory cytokines and bone markers were evaluated.

## 2. Materials and Methods

### 2.1. Synthesis and Characterization of the Apatitic Powders

Hydroxyapatite (HA) and hydroxyapatite-zoledronate (HA-ZOL) crystals were prepared by direct synthesis in aqueous solution in N_2_ atmosphere. For the synthesis of HA, 50 mL of 0.65 M (NH_4_)_2_HPO_4_ solution was added dropwise under stirring to 50 mL of 1.08 M Ca(NO_3_)_2_ 4H_2_O solution at pH adjusted to 10 with NH_4_OH at 90 °C. Stirring was maintained for 5 h at 90 °C, then HA was centrifuged at 10,000 rpm for 10 min and washed with CO_2_-free distilled water. HA-ZOL was obtained by adding disodium zoledronate tetrahydrate (Chemos, GmbH, Altdorf, Germany) to the phosphate solution. The amount of Zoledronate was calculated on final volume to 20 mM.

X-ray diffraction analysis was carried out by means of a PANalytical X’Pert PRO (Malvern PANalytical, Milano, Italy) powder diffractometer equipped with a fast X’Celerator detector (40 mA, 40 kV). For phase identification, the 2θ range was investigated from 10 to 50 2θ degrees with a step size of 0.1° and time/step of 100 s. For Transmission Electron Microscopy (TEM) investigations, a small amount of powder was dispersed in ethanol and submitted to ultrasonication. A drop of the calcium phosphate suspension was transferred onto formvar films supported on conventional copper microgrids. A Philips CM 100 TEM operating at 80 kV was used. Zoledronate content was determined spectrophotometrically via complex formation with Fe(III) ions using a Varian Cary50Bio (Agilent Technologies Italia SpA, Milano, Italy) instrument (λ = 290 nm).

### 2.2. Scaffolds Preparation and Characterization

Type A gelatin from porcine skin (250 Bloom, Sigma Aldrich, Milano, Italy) and genipin (Wako Chemicals, Neuss, Germany) were used. Composite scaffolds were obtained following the procedure previously reported [24]. Briefly, 15 g of gelatin and 6.4 g of HA or HA-ZOL were poured into a flask containing 130 mL of distilled water. The mixture was maintained at 55 °C under gentle stirring until gelatin dissolution, and then foamed by mechanical stirring at 600 rpm for about 5 min. Gelatin crosslinking was obtained through addition of 10 mL of an aqueous genipin solution (0.15% wt/V) and 10 mL of a phosphate buffered solution (1 M at pH 7.4) to the foam. After just a few minutes, the foam was deposited on Petri dishes (diameter = 9 cm) and allowed to gelify at 37 °C for 3 h. Then, the samples were rinsed in a 0.1 M Glycine aqueous solution, then washed in distilled water and immersed in ethanol for 24 h. Scaffolds were obtained after freeze drying for 12 h at −44 °C and 0.45 mbar and labelled GHA and GHAZOL. The morphology of the scaffolds was analyzed with a Philips XL-20 Scanning Electron Microscope operating at 15 kV on samples, which were sputter-coated with Pd prior to examination. Prior to in vitro experiments GHA and GHAZOL samples were soaked in ethanol 70% solution, UV irradiated overnight and extensively washed in phosphate buffered saline (PBS, Gibco, Fisher Scientific Italia, Milano, Italy).

### 2.3. In Vitro Fracture Model

**Figure 1:**
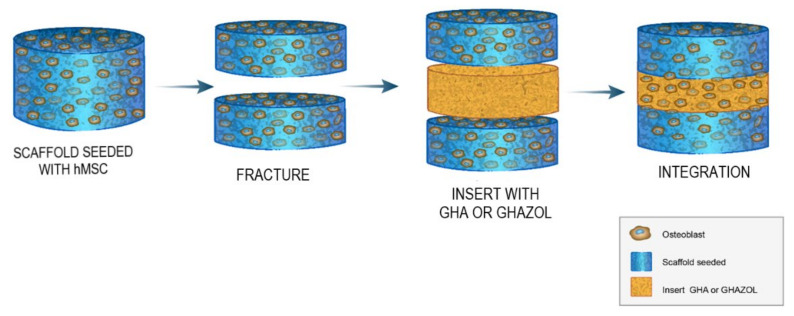
Study design. In vitro fracture model adopted by Sundelacruz et al. [23]: unseeded GHA or GHAZOL scaffolds are inserted between the two halves of scaffold cultured for three days with hMSCs differentiated into osteoblastic lineage to evaluate cell morphology, viability, osteogenic differentiation extracellular matrix deposition and the presence of inflammatory cytokines at the selected experimental times of 3, 7 and 10 days.

Normal human bone-marrow derived mesenchymal stem cells (hMSC, PCS-500-012, lot. n 8778, ATCC, USA) were expanded in Basal Medium (MSC-BM, ATCC). After expansion, cells were counted and seeded at concentration of 5 × 10^4^ cells/mL in 24-well plates containing sterile samples of GHA (T0 experimental time) and in monolayer controls (CTR). Two different 3D controls were also selected: GHA seeded with cells in Osteogenic Differentiation Medium (MSC-DM, ATCC) (differentiated control, CTRd) and in basal MSC-BM medium (undifferentiated control, CTRnd). Culture plates were maintained in standard conditions (5% CO_2_ and 37 °C) for 3 days. Then, the 3D fracture model was created: seeded GHA scaffolds were half-cut to simulate fracture and GHA or GHAZOL scaffolds were interposed (T1 experimental time, Figure 1). Assembled 3D models were cultured in the above reported standard conditions for 10 days and medium changed twice a week.

### 2.4. Cell Viability and Morphology

Evaluations were carried out at 3, 7, and 10 days to assess viability and morphology of hMSC in the 3D fracture model and CTRs. Viability was evaluated by Live/Dead^®^ assay (Molecular Probes, Eugene, OR, USA), according to the manufacturer’s instructions. Briefly, 3D samples were rinsed in PBS and incubated with 2 μM Calcein AM and 4 μM EthD-1 for 45 min in the dark at room temperature. Then, samples were visualized using an inverted microscope equipped with an epifluorescence setup excitation/emission setting of 488/530 nm to detect green fluorescence (live cells) and 530/580 nm to detect red fluorescence (dead cells); images were captured at 4 and 10× magnification. Cell morphology was observed through SEM investigation. Cells grown on the materials were fixed with glutaraldehyde (2.5%) in 0.01 M phosphate buffer (pH 7.4) for 1 h. After dehydration in a graded ethanol series, the samples were immersed in hexamethyldisilazane (Merck KGaA, Darmstadt, Germany) and then air dried. The samples were mounted on Al stubs and sputter-coated with Pd prior to examination with a Philips XL-20 Scanning Electron Microscope operating at 15 kV.

### 2.5. Cell Differentiation and Metabolic Activity

#### 2.5.1. Gene Expression by RT-PCR

Gene expression analysis of the most common markers of osteoblastic differentiation was performed on all groups at 3, 7 and 10 days. Total RNA was extracted from all samples at each experimental time using PureLink RNA Mini Kit (Life Technologies, Carlsbad, CA, USA) and reverse transcribed with SuperScript VILO cDNA Synthesis Kit (Life Technologies, Carlsbad, CA, USA), following the manufacturer instructions. A semi-quantitative polymerase chain reaction (PCR) analysis was performed for each sample in duplicate in a LightCycler 2.0 Instrument (Roche Diagnostics GmbH, Manheim, Germany) using QuantiTect SYBR Green PCR Kit (Qiagen, Hilden, Germany) and gene-specific primers (Table 1). After a melting curve analysis to check for amplicon specificity, the threshold cycle was determined for each sample and relative gene expression was calculated using the ^2−ΔΔ^Ct method [25]. For each gene, expression levels were normalized to *GAPDH* (Glyceraldehyde 3-phosphate dehydrogenase) using undifferentiated cells (CTRnd) for each experimental time as calibrators.

#### 2.5.2. Immunoenzymatic Assays

Proinflammatory cytokines and protein analysis of main bone markers produced by cells within the 3D model were measured by ELISA colorimetric tests for the quantification of Interleukin 6 (IL6, SEA079Hu Cloud-Clone Corp, USA), Interleukin 1ß (IL1ß, EK0392, Boster Bio, Pleasanton, CA, USA), Tumor Necrosis Factor α (TNFα, SEA133Hu, Cloud-Clone Corp., Katy, TX, USA), Collagen alpha-1(I)chain (COLL1a1, SEA350Hu Cloud-Clone Corp., Katy, TX, USA) and Alkaline Phosphatase (ALP, SEB472Hu Cloud-Clone Corp., Katy, TX, USA). Supernatants were collected from all wells at all experiment times and maintained at −20 °C until used.

### 2.6. Statistical Analysis

Statistical evaluation of data was performed using the software package SPSS/PC^+^ Statistics ^TM^ 25.0 (SPSS Inc., Chicago, IL, USA). The results are presented as the mean of six independent values ± standard deviations (SD) at a significance level of *p* < 0.05. After having verified non-normal distribution and homogeneity of variance, a Tamhane *post hoc* test was applied to detect significant differences among groups.

## 3. Results

The 3D in vitro model developed in this work involves the use of composite scaffolds, GHA, which was seeded with hMSC. After three days, the scaffolds were cut in halves and a further slice of unseeded scaffold was inserted between the two halves of the original one. The insert scaffold contained zoledronate functionalized hydroxyapatite (GHAZOL), whereas a slice of GHA was used as an insert for control samples.

### 3.1. Scaffolds Characterization

**Figure 2:**
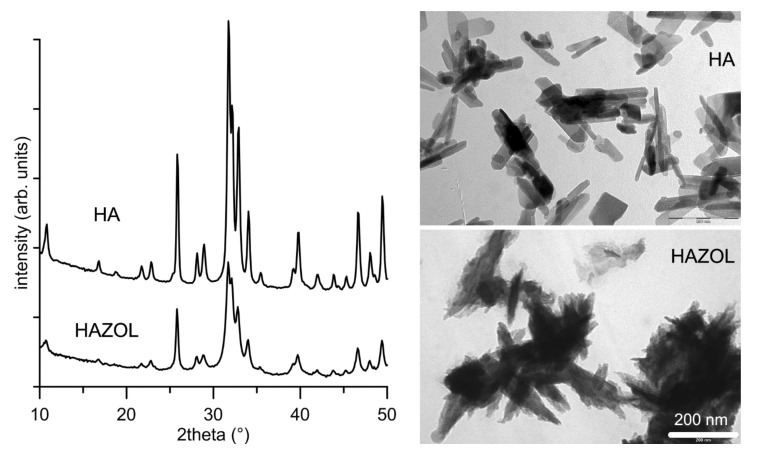
Powder X-ray diffraction patterns and TEM images of HA and HAZOL nanocrystals. Scale bar (200 nm) is common for the two images, for direct comparison. arb. = arbitrary.

The inorganic phase content of both GHA and GHAZOL was 30 wt%. The comparison of the powder X-ray diffraction patterns of HA and HAZOL reported in Figure 2 shows that both materials contain a single crystalline phase, hydroxyapatite (PDF n. 9-432). However, the XRD peaks present in the pattern of HAZOL appear slightly broadened in comparison to those of HA, suggesting that the presence of the bisphosphonate provokes a slight reduction in the degree of crystallinity. Moreover, the dimensions of HAZOL nanocrystals are smaller and their shape worse defined than those of HA nanocrystals, as it can be observed in the TEM images reported in Figure 2.

The amount of the bisphosphonate in HAZOL nanocrystals, which was evaluated spectrophotometrically through analysis of its chromophoric complex with Fe(III) ions [26], was 10.9 ± 0.5 wt%.

**Figure 3:**
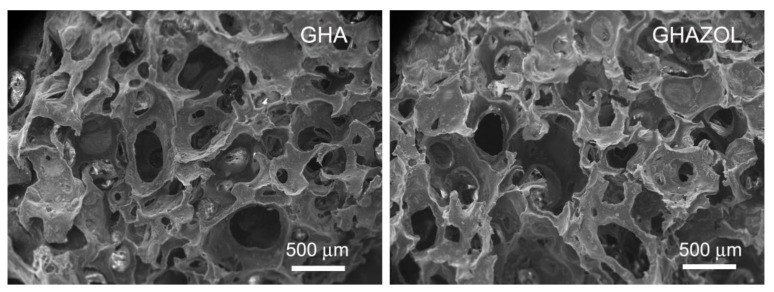
SEM micrographs of the cross-sections of the composite scaffolds: GHA and GHAZOL show porous structures, with no significant difference. Scale bar (500 nm) is common for the two images, for direct comparison. Magnifications = 30×.

The method utilized for the preparation of the scaffolds, which implies foaming of the gelatin solution containing a suspension of the inorganic phase, crosslinking with genipin, gelling and freeze-drying, provided porous materials. In fact, SEM images of the cross-sections of the scaffolds show the presence of numerous, interconnected pores through the scaffolds, with no appreciable differences between GHA and GHAZOL (Figure 3).

### 3.2. Cells Viability and Morphology

**Figure 4:**
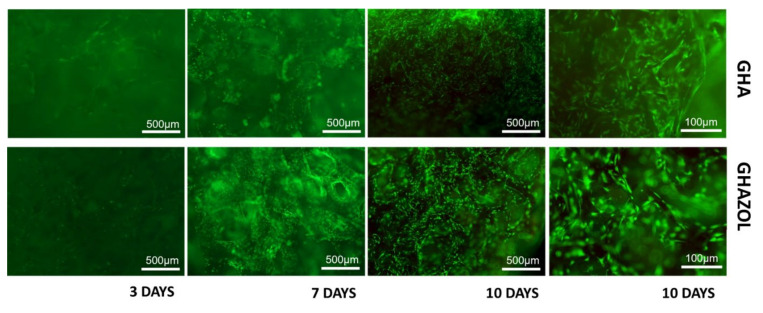
Viability of materials. Live and dead cell staining was conducted on differentiated hMSC in the 3D fracture models treated with GHA and GHAZOL after 3, 7 and 10 days of culture. Viable cells stain green while dead cells stain red: images show that cells grew regularly into both 3D porous scaffolds as evident by the green fluorescent dye in absence of the red fluorescence dye (4× magnification: bar = 500 μm; 10× magnification: bar = 100 μm).

The colonization of GHA and GHAZOL inserts by differentiated hMSC was evaluated to assess initial phases of implant integration and fracture healing. Cell viability and morphology were observed after Live/Dead^®^ assay (Figure 4): the merged images showed that hMSC were viable and proliferated regularly on both GHA and GHAZOL 3D porous scaffolds. It could be observed by fluorescent dye, at 4× of magnification, that at 3 days, only few cells migrate into the insert, but at 7 and more at 10 days, cells progressively colonized both GHA and GHAZOL scaffolds. At 10 days, higher magnification showed cells inside the scaffold’s porosities.

**Figure 5:**
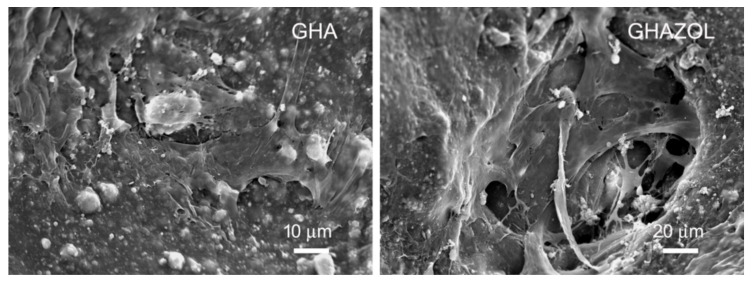
SEM micrographs of hMSC grown after 10 days of culture on the inner layers of GHA and GHAZOL inserts. Scale bars: 10 and 20 μm for GHA and GHAZOL, respectively. Magnifications: 1000× and 500× for GHA and GHAZOL, respectively.

SEM images confirm the presence of cells in the inner layers of the scaffolds: cells appeared well spread and rich with filopodia independently from the nature of the inorganic phase (GHA or GHAZOL) present in the inner layer (Figure 5). These results demonstrated that scaffolds not only support cell growth, but cells were also able to adhere, proliferate and colonize the whole scaffolds.

### 3.3. Cell Differentiation and Metabolic Activity

#### 3.3.1. Gene Expression by RT-PCR

**Figure 6:**
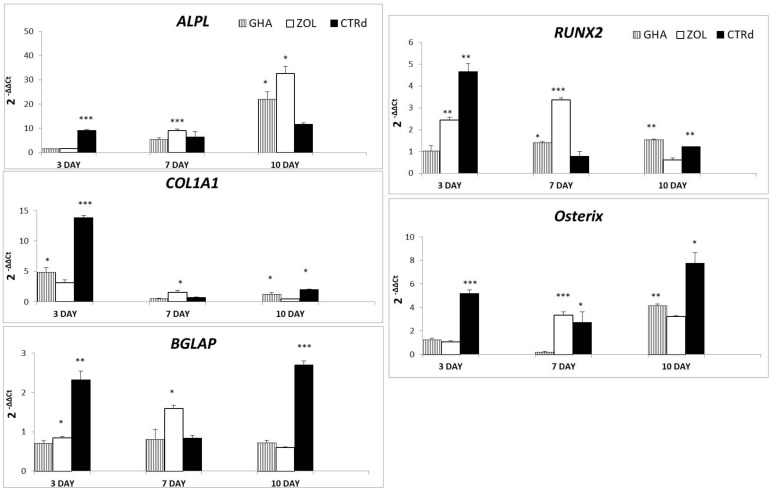
Realtime PCR results of the major osteoblastic lineage genes; hMSC were cultured on GHA and GHAZOL 3D fracture model and on CTRd for 3, 7 and 10 days, thereafter RNA was extracted and the relative gene expression of *ALPL*, *COL1A1*, *BGLAP*, *RUNX2* and *OSTERIX* was calculated by normalizing to a housekeeping gene (*GAPDH*)(*: *p* < 0.05; **: *p* < 0.005, ***: *p* < 0.0005).

All samples were evaluated at 3, 7 and 10 days of culture investigating the expression of the most common genes related to osteoblast activity and differentiation. In detail, *ALPL*, *COL1A1* and *BGLAP* genes (Figure 6) are representative of osteoblast activity for the production of alkaline phosphatase, collagen type I and osteocalcin, as typical products of bone extracellular matrix. *ALPL* expression of cells on biomaterials increased from very low level at 3 days to progressively and significantly higher values at 7 (GHAZOL, *p* < 0.0005) and 10 days (GHA and GHAZOL, *p* < 0.05) when compared to CTRd. Moreover, at 7 days GHAZOL value was significantly higher than GHA (*p* < 0.0005). As well as *ALPL*, *COL1A1* showed a higher expression in CTRd than in GHA and GHAZOL (CTRd, *p* < 0.0005) at 3 days, while the expression significantly increased over CTRd at 7 days (GHAZOL, *p* < 0.05). At 10 days, both GHA and CTRd showed higher expression in comparison to GHAZOL (*p* < 0.05). *BGLAP* expression in GHA and GHAZOL also increased from values lower of CTRd at 3 days (CTRd, *p* < 0.005) to increased levels of expression at 7 days, then *BGLAP* was downregulated again at 10 days in comparison to CTRd (CTRd, *p* < 0.0005). GHAZOL was significantly higher than GHA at 3 days (GHAZOL, *p* < 0.05) and GHA and CTRd at 7 days (GHAZOL, *p* < 0.05).

Both *RUNX2* and *OSTERIX*, genes representative of osteoblastic differentiation, are reported in Figure 6. Results of CTRd demonstrated that hMSC were able to differentiate toward osteoblastic lineage since all values, although the experimental times were higher than CTRnd (data not shown). Regarding *RUNX2*, GHAZOL and CTRd showed higher value (*p* < 0.005) in respect to GHA at 3 days. At 7 days, GHAZOL increased significantly over CTRd and GHA (*p* < 0.0005) and GHA significantly over CTRd (GHA, *p* < 0.05). At 10 days, CTRd and GHA showed increased levels of expression than GHAZOL (CTRd and GHA, *p* < 0.005). *OSTERIX* was highly expressed in CTRd at 3 (*p* < 0.0005) and 10 days (*p* < 0.05), while GHAZOL reached the highest expression at 7 days (GHAZOL, *p* < 0.0005) and GHA at 10 days (GHA, *p* < 0.005).

#### 3.3.2. Immunoenzymatic Assays

**Figure 7:**
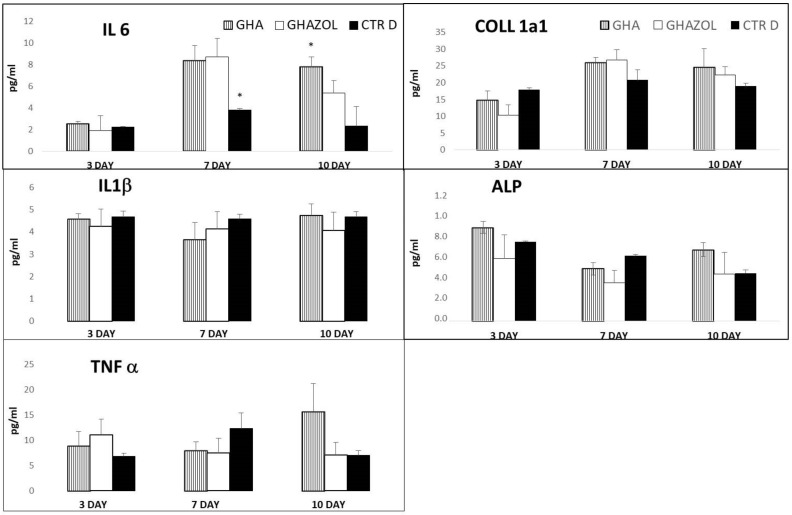
Cytokine and bone-related protein release. Evaluation of pro-inflammatory growth factors, Interleukin 6 (IL6), Interleukin 1 beta (IL1β), Tumor Necrosis Factor alpha (TNFα) and main bone markers, Collagen alpha-1(I)chain (COLL1a1) and Alkaline Phosphatase (ALP) secreted by the 3D fracture models in the culture medium and assayed by immunoenzymatically tests after 3, 7, and 10 days of culture. Data are reported as mean±standard deviation (*: *p* < 0.05).

Levels of secreted pro-inflammatory cytokines, IL6, IL1β and TNF-α, and main bone markers, COLL1a1 and ALP, are shown in Figure 7.

The evaluation of IL6 showed an increased significant level at 7 days in GHA and GHAZOL in comparison with CTRd (*p* < 0.05). GHA maintained such increased significant values also at 10 days (*p* < 0.05). The IL6 produced by hMSC in GHAZOL was found to be transiently high at 7 days, with respect to 3 and 10 days, in which no significant differences with CTRd were detected. IL1ß and TNFα were not affected by both GHA and GHAZOL scaffolds and no significant differences were found among them and CTRd. COLL1a1 was more expressed after 7 days of culture, while ALP, as early bone marker, was higher at 3 days. Both protein synthesis secreted by hMSC into GHA and GHAZOL showed similar results as in CTRd, with no significant differences.

## 4. Discussion

The objective of the study was the assessment of the capacity of an innovative porous scaffolding biomaterial to promote cell adhesion and viability to maintain the osteogenic differentiation status and to bio-actively promote the expression of anabolic markers linked to the bone extracellular matrix deposition. Moreover, by developing an in vitro model of wound healing, we aimed at increasing the tridimensional complexities to evaluate its capacity in a 3D model of fracture healing relevant for bone regenerative purposes.

The overall observation of data revealed that hMSC analyzed within the inserts were fully viable in the absence of dead cells. SEM images showed a uniform and continuous cell layer spread onto the porous surfaces of the scaffolds, demonstrating that both in GHA and GHAZOL were able to support cell growth, adhesion and progressively colonize the whole inserts in absence of any cytotoxic effects. From a molecular point of view, we next quantified the gene expression of main genes involved in the osteogenic commitment and differentiation of hMSCs, and in their metabolic activity, focused on the secretion of bone extracellular matrix. Firstly, results showed that, at 7 days of culture within the 3D model, the expression of the analyzed genes was enhanced over the monolayer cell culture (CTRd). In particular, the comparison between the two scaffolds demonstrated a significantly higher expression of *ALPL*, *COL1A1*, *BGLAP*, *RUNX2*, *Osterix* genes in GHAZOL at 7 days when compared to GHA. These results confirmed that GHA and GHAZOL scaffolds sustain not only cell proliferation, but positively influence osteoblast differentiation and activity if employed in fracture healing. Then, we aimed to determine if this fracture model could mirror the inflammatory response that is part of the first phase of the healing process that occurs in humans typically 1–5 days after a bone fracture [6] and leads to a local inflammation with the role of MSC activation and migration via autocrine and paracrine pathways by the release of pro-inflammatory cytokines [27]. For this aim, we quantified in the supernatants the levels of the main secreted pro-inflammatory cytokines and main bone markers. While ALP and COLL1a1 protein levels were maintained, we found that the 3D fracture model healed by the insertion of GHA or GHAZOL could induce a transient increase at 7 days of IL6 in comparison with the 2D monolayer culture. High values of IL6 are generally related to its involvement in bone remodeling. Despite chronic high levels of IL6 having a negative effect on bone microenvironment, a transient enhancement of IL6, as evidenced in our study, plays an important role in promoting osteogenic commitment and stimulating bone regeneration, also acting as a modulator of other cytokines with paracrine signaling [28,29].

In general, promising scaffold materials for bone regenerative purposes should be biocompatible, osteoconductive and osteoinductive, meaning that they should induce cell attachment, proliferation and differentiation towards osteogenic phenotype and conduct new bone apposition in absence of toxic or adverse reaction [30]. Functionalization with bioactive agents, such as antiosteoporotic drugs to locally increase the drug bioavailability and counteract bone resorption, might improve the biological performance of innovative scaffolding materials [18].

In this scenario of newly developed materials, an innovative in vitro model of bone fracture healing might be a promising tool to be used as a prescreen tool for assessing a candidate scaffold for bone regeneration, to reduce or refine animal experimentation. In the present study, this model allowed to characterize some features of the tested innovative scaffolds even combined with antiosteoporotic drug; hMSC adhesion, viability and differentiation on GHA and GHAZOL were determined confirming the absence of cytotoxicity and their biocompatibility. Moreover, the quantification of expression of the main genes involved in extracellular matrix deposition revealed the bioactive nature of scaffolds capable of inducing anabolic cell pathways. The 3D fracture model recapitulated the first phase of healing in which a transient inflammatory response is needed to stimulate regenerative processes.

In vitro strategies, developed and adopted by researchers to evaluate biomaterial efficacy in terms of cytotoxicity, bioactivity, osteoinductive and osteoconductive properties, usually employ the seeding of a single cell phenotype on the biomaterial. Improvements, aimed at increasing in vitro complexities are represented by co- and tri-cultures onto biomaterials that have been used to recapitulate the adhesion and differentiation phases of several phenotypes that concur and exert their role at the bone-biomaterial interface [31,32]. The use of co-cultures with the combination of inflammatory cells, mesenchymal stem cells, osteoblasts, osteoclasts or endothelial cells might be useful to mimic the in vivo bone microenvironment. The behaviour of single cultures demonstrated to be different compared to the bi- or tri-cultures and depending on the cell types involved [31]. It was also demonstrated that an innovative in vitro model which involves the tri-culture of osteoblasts, osteoclasts and endothelial cells suggests that some biomaterials can exert a beneficial action onto bone repair microenvironment, stimulating osteoblast proliferation and activity, downregulating osteoclastogenesis and supporting microangiogenetic processes necessary for new bone formation [32]. Recent advances in the cross talk between different cell phenotypes included the development of micro fluidic devices, which provide the permanent supply of nutrients with the monitoring of pH, temperature and oxygen; with the set of multi-chambers, the microfluidic systems and their miniaturization called organ-on-a-chip platforms might combine serially two or more phenotypes [6] In addition, in silico models have been recently developed as an alternative to in vivo experimentations: advances in bioinformatics have enabled the modelling, simulation and prediction of some cellular processes and diseases by describing them mathematically through computational models. These simplified models are to be considered complementary (and to not replace) experimental research, since they lack the simulation of biological response [33,34,35].

From an ethical and legal point of view, the adoption and validation of advanced 3D in vitro models are mandatory to avoid or, at least, reduce the need of in vivo models. In fact, beginning from the Russell and Burch 3R principles on the replacement, reduction and refinement of animals used for scientific purposes and in compliance with the directive 2010/63/EU, the number of animals for in vivo studies should be reduced to a minimum or replaced by advanced alternative in vitro and ex vivo models [36,37]. For these reasons, there is an urgent need to explore and establish reliable in vitro innovative models to widen the options of available tools for the screening of biomaterials before the adoption of in vivo models and clinical studies.

Limitations of the present study are: (i) the lack of longer experimental times that could have allowed the determination of the following phases of bone fracture healing: fibrocartilaginous callus formation, bony callus secretion through vascularization and mineralization and finally bone remodeling phases towards the formation of a mature lamellar bone [38]; (ii) the lack of mechanical stimulus, although most fractures are mechanically stabilized; and (iii) the lack of a pathological microenvironment by means of adopting cells harvested from osteoporotic patients. In fact, in vitro models that adopt cells and/or tissues from osteoporotic patients have been shown biological differences in cell self-renewal ability, differentiation, proliferation, cytokine and growth factor productions [39,40,41,42,43]. Further studies performed with cells harvested from pathological osteoporotic patients are also needed to confirm the efficacy of the scaffold doped with zoledronate in boosting the osteogenic and regenerative properties in misbalanced pathological cells. In addition, the close connection among in vitro, in silico, ex vivo and in vivo models and not the rigid separation is of uttermost importance to collect data and find the way towards a better understanding and development of new therapies.

## 5. Conclusions

This study usefully employed an advanced in vitro 3D model of healing to investigate biological properties of innovative porous scaffolds for bone tissue engineering or regenerative purposes. Hydroxyapatite nanocrystals alone and even doped with zolendronate were able to promote human mesenchymal stromal cell adhesion, viability, differentiation towards osteogenic phenotype. Cells colonizing empty scaffolds inserted in the in vitro wound healing model maintaining viability and metabolic activity. These properties of a material to maintain mesenchymal phenotype, to stimulate osteogenesis and colonization are relevant for bone regenerative purposes even in cases of alterations of bone metabolism such as osteoporosis where the presence of locally delivered zoledronate might enhance its local bioavailability contributing to the rebalance of the bone remodelling.

## Figures and Tables

**Table 1:** Primer sequences.

Symbol	Gene	Primer FW	Primer RV	T Annealing	Amplicon Length
*ALPL*	Alkaline phosphatase liver/bone/kidney	QT00012957 *		55 °C 20′′	110 bp
*COL1A1*	Collagen type 1, chain a 1	QT00037793 *		55 °C 20′′	118 bp
*BGLAP*	Osteocalcin	QT00232771 *		55 °C 20′′	90 bp
*RUNX2*	Runt related transcription factor 2	CTTCACAAATCCTCCCCAAGT	AGGCGGTCAGAGAACAAAC	60 °C 20′′	212 bp
*SP7*	Osterix	QT000213514 *		55 °C 20′′	120 bp
*GAPDH*	Glyceraldehyde-3-phosphate dehydrogenase	TGGTATCGTGGAAGGACTCA	GCAGGGATGATGTTCTGGA	56 °C 20′′	123 bp

* QuantiTect Primer Assay (Quiagen).

## Data Availability

The data that support the findings of this study are available from the corresponding author upon reasonable request.
